# COMPREHENSIVE CARE FOR JOINT REPLACEMENT MODEL: POST-ACUTE CARE AND PREFERRED PROVIDER NETWORKS

**DOI:** 10.1093/geroni/igz038.3172

**Published:** 2019-11-08

**Authors:** Cynthia A Gruman, Amy Cowell, Kathryn Palmisano, Shannon Rogers, Laura Dummit

**Affiliations:** 1 The Lewin Group, Falls Church, Virginia, United States; 2 The Lewin Group, Eden Prairie, Minnesota, United States

## Abstract

The Comprehensive Care for Joint Replacement (CJR) model, implemented by the Centers for Medicare & Medicaid Services in 2016, is a randomized, controlled trial that tests the effect of holding a hospital accountable for payments and quality of all services provided to lower extremity joint replacement (LEJR) patients during an episode of care. The newly released results include 147,923 LEJR episodes that were initiated by 733 hospitals in 67 randomly selected metropolitan statistical areas. The objective of this presentation is to explore changes to the care pathway using results from a mixed-methods analytic approach including triangulation of findings from analysis of Medicare claims, hospital survey and hospital and associated provider interview data. Hospitals reported implementing notable changes over the past two years including hiring navigators, changes to therapy protocols, and direct discharge home. Hospital interviewees described efforts to strengthen relationships with PAC providers including the investment of resources into the development of preferred PAC provider networks. As a result of these changes, the average number of SNF days decreased by 2.3 days more for CJR episodes than for control group episodes from the baseline to the intervention period (p<0.01). Changes in two of nine complexity measures indicated a statistically significant relative decrease in CJR patients’ functional status at SNF admission. The relative increases in CJR patients’ average early-loss activities of daily living (ADLs) scores (p<0.05) and motion scores (p<0.10) suggest an increase in patients with greater needs were discharged to a SNF relative to the control group.

